# GUTSS: An Alignment-Free Sequence Comparison Method for Use in Human Intestinal Microbiome and Fecal Microbiota Transplantation Analysis

**DOI:** 10.1371/journal.pone.0158897

**Published:** 2016-07-08

**Authors:** Mitchell J. Brittnacher, Sonya L. Heltshe, Hillary S. Hayden, Matthew C. Radey, Eli J. Weiss, Christopher J. Damman, Timothy L. Zisman, David L. Suskind, Samuel I. Miller

**Affiliations:** 1 Department of Microbiology, University of Washington, Seattle, Washington, United States of America; 2 Department of Pediatrics, University of Washington, Seattle, Washington, United States of America; 3 Seattle Children's Research Institute, Seattle, Washington, United States of America; 4 Division of Gastroenterology, University of Washington, Seattle, Washington, United States of America; 5 Seattle Children’s Hospital, Seattle, Washington, United States of America; 6 Department of Medicine, University of Washington, Seattle, Washington, United States of America; 7 Department of Immunology, University of Washington, Seattle, Washington, United States of America; 8 Department of Genome Sciences, University of Washington, Seattle, Washington, United States of America; National Cancer Institute, UNITED STATES

## Abstract

**Background:**

Comparative analysis of gut microbiomes in clinical studies of human diseases typically rely on identification and quantification of species or genes. In addition to exploring specific functional characteristics of the microbiome and potential significance of species diversity or expansion, microbiome similarity is also calculated to study change in response to therapies directed at altering the microbiome. Established ecological measures of similarity can be constructed from species abundances, however methods for calculating these commonly used ecological measures of similarity directly from whole genome shotgun (WGS) metagenomic sequence are lacking.

**Results:**

We present an alignment-free method for calculating similarity of WGS metagenomic sequences that is analogous to the Bray–Curtis index for species, implemented by the General Utility for Testing Sequence Similarity (GUTSS) software application. This method was applied to intestinal microbiomes of healthy young children to measure developmental changes toward an adult microbiome during the first 3 years of life. We also calculate similarity of donor and recipient microbiomes to measure establishment, or engraftment, of donor microbiota in fecal microbiota transplantation (FMT) studies focused on mild to moderate Crohn's disease. We show how a relative index of similarity to donor can be calculated as a measure of change in a patient's microbiome toward that of the donor in response to FMT.

**Conclusion:**

Because clinical efficacy of the transplant procedure cannot be fully evaluated without analysis methods to quantify actual FMT engraftment, we developed a method for detecting change in the gut microbiome that is independent of species identification and database bias, sensitive to changes in relative abundance of the microbial constituents, and can be formulated as an index for correlating engraftment success with clinical measures of disease. More generally, this method may be applied to clinical evaluation of human microbiomes and provide potential diagnostic determination of individuals who may be candidates for specific therapies directed at alteration of the microbiome.

## Introduction

Research studies seeking to establish the potential role of the gut microbiome in human disease have been greatly aided by advances in sequencing technologies and the parallel development of sequence analysis methods. Standard methods of microbiome analysis have relied upon identification of species as a basis for inferring characteristics of the microbiome that may be associated with disease. The interpersonal variability of bacterial species in the human gut microbiome suggests that the gene or functional complement of the microbial community may be more relevant than species alone when analyzing differences in the microbiome associated with disease[1]. In addition to identifying compositional patterns of microbial communities or the complement of metabolic functions to correlate characteristics of microbiomes with disease, there is also value in determining ecological resemblance through quantitative measures such as similarity or dissimilarity (β-diversity). Similarity or difference measures have been widely discussed in the fields of ecology, biology and biogeography[2] and are often derived from species counts or relative abundance[3, 4]. The UniFrac[5] distance is derived from phylogenetic tree branch lengths between species, sometimes weighted by species abundances, based on the concept that closely-related organisms may share functional characteristics in common. Because similarity and dissimilarity measures are typically calculated from species abundance estimates, they are biased by the methods used to identify species and estimate abundances.

A widely used method of identifying and quantifying species or operational taxonomic units (OTUs) is targeted amplicon, 16S rRNA gene sequencing[6, 7]. Benefits of this approach are that the 16S rRNA gene is universal in bacteria, representation of the 16S rRNA gene sequence in GenBank is extensive, sequencing a short (~1500 bp) region is relatively inexpensive, and common use of the same target gene across multiple studies increases accuracy in meta-analysis[8]. Although its variable regions can be used to distinguish taxa it provides uneven resolution of the taxonomic spectrum, and variation in copy number of this gene influences abundance estimates, among other limitations[9]. Microbial abundance profiles can also be generated from WGS metagenomic sequence by methods that include alignment, taxonomic classification, metagenomics assembly, binning and deconvolution. Relative abundances can be estimated by aligning WGS metagenomic sequence to taxon specific marker genes[10], unique genomic regions[11], complete genomes[12–14] and universal marker genes[15]. Homology-based methods have also been introduced to correct abundance estimates from alignment using a variety of modeling strategies[16–18]. Accuracy of the abundance estimates using alignment methods has improved with increasing representation of taxa in reference databases. However, this approach will always be limited by ability to identify microbial strains that are distant from the reference and those which are modified by gene acquisition or deletion as they adapt to new environments and compete for resources. Some of these adaptations may be associated with the particular human disease being investigated. Taxonomic classification of individual WGS metagenomic sequence reads to group them with the assistance of phylogenetic relatedness can improve identification of novel strains[19, 20]. Classification methods can be biased by uneven and limited representation of taxa and the scarcity of unculturable microbes in reference databases. These limitations can be overcome by metagenomic assembly[21–24], given sufficient read coverage, especially when combined with other methods of determining microbial abundances. Binning or partitioning of the sequence reads using frequency patterns of *k*-mers, short nucleotide (nt) sequences of length *k*, provides a means of clustering WGS metagenomic sequence that is independent of reference databases[25–28]. The use of subtle differences in frequency patterns for non-unique *k*-mers can help distinguish reads from different taxa but this method has relatively lower resolution, especially for closely related species. Deconvolution methods[29–31] invert high-dimensional matrices to resolve the relationship between gene content or function and taxonomic abundances. This is a multi-sample analysis method that is dependent on the number and content of the metagenomic samples for accuracy. At the current stage they are also dependent on reference databases and annotation, but are less sensitive to incompleteness of these resources.

An alternative approach to measuring microbiome similarity that is independent of species abundance estimation and reference databases is direct comparison of metagenomic shotgun sequence reads using "alignment-free" methodology[32–34]. The advantage of alignment-free methods is that they avoid incomplete database bias, OTU identification error, inability to assemble low coverage (low abundance) OTUs and they are robust even for unculturable microbes. An efficient method of determining sample similarity in WGS metagenomic sequence is through the use of *k*-mers. By direct comparison of metagenomic sequence at the scale of shotgun sequence reads, similarity can be measured at a subspecies level. The dissimilarity or distance measures derived from them can be used for classification by clustering, construction of phylogenetic-like tree graphs, or ordination such as principal coordinates analysis[1, 35]. The disadvantage of these methods is that the derived scores are not standard ecological measures. Quantitative measures of ecological similarity or distance derived from species identification and abundance such as UNIFRAC[36–38], Jensen-Shannon[39, 40], Bray-Curtis (BC)[39, 41–44] and Morisita-Horn[45] are commonly used for microbiome analysis in clinical research studies. However, methods to calculate these measures from WGS metagenomic sequence using alignment-free algorithms are lacking. The alignment-free method described in this report, which estimates microbiome similarity with a metric that is analogous to the BC ecological measure, fills this gap in methodology.

Our method was motivated by the need for accurate estimation of sample similarity in paired comparison of WGS metagenomic sequence-derived microbiomes before and after treatment to evaluate new therapies in clinical studies. For example, successful treatment of recurrent *Clostridium difficile*[38–40, 42, 45, 46] induced colitis with fecal microbiota transplantation (FMT) has stimulated research in evaluating the effectiveness of FMT as therapy for ulcerative colitis (UC)[37, 44], Crohn's disease (CD)[47] and other inflammatory bowel diseases (IBD)[41, 43, 48, 49], which may have a microbial component to the disease. This therapy aims to replace the gut microbiota in a recipient by colonic or nasogastric administration of filtered and diluted stool obtained from a healthy donor. FMT is hypothesized to work by shifting the recipient's microbiota toward a community that resists inflammation induced by pathogens or an altered microbiota. Though case reports indicate episodic success of these therapies for IBD, there has been limited analysis of engraftment performed in these studies to assess the therapeutic potential of FMT to alter the microbiome in a sustained manner[36]. We sought to develop a method to measure the extent to which the donor's gut microbiota has been established in the recipient following transplantation based on similarity of the microbiomes of the patient and donor. In a recent pediatric study to treat Crohn's disease with FMT at Seattle Children's Hospital[47] we applied an alignment-free algorithm, Compareads[50], more recently released as Commet[51], to measure engraftment using WGS metagenomic sequence. Commet is a computationally efficient algorithm that calculates similarity on the basis of sequence identity, which is determined by mapping *k*-mers between samples. However, it is not a standard ecological measure and its estimate of similarity can be greatly inflated for samples with high asymmetry of abundance since it does not take relative sequence abundance into account.

Here we report the development of an alignment-free algorithm, GUTSS that takes into account differences in abundance in a manner analogous to the BC index[52] to measure similarity of microbiomes using WGS metagenomic sequence. While alignment-free algorithms count *k*-mers[34], use compression[53] or other methods to calculate similarity or dissimilarity[32], we followed the same general approach as Commet in counting reads, identified by matching *k*-mer sequences, that overlap in two metagenome samples. In contrast to Commet, which tallies all reads that map between the two samples, we use differences in *k*-mer counts in shared reads to account for relative abundance of sequences in the two samples. We present a description of the methodology and results from applying the method to microbiome data from two clinical studies. Longitudinal samples from healthy young children participating as controls in a pediatric research study of cystic fibrosis (CF) at Seattle Children's Hospital[54] were compared with adults in the Human Microbiome Project (HMP)[55] to measure developmental changes toward an adult microbiome during the first three years of life. An analysis of the microbiome data from a pediatric study to treat Crohn's disease with FMT at Seattle Children's Hospital[47] is also presented to show how this methodology is particularly valuable for evaluating specific therapies directed at altering the microbiome such as FMT to treat IBD.

## Materials and Methods

### Similarity score

One of the most well-known coefficients of ecological resemblance is the *percentage difference*, which in its one-complement form is commonly referred to as the Bray–Curt is index of similarity[2]. The BC index of percent similarity can be expressed as[56]

$$S=100\frac{\sum_{i}\text{min}(X_{i},Y_{i})}{\sum_{i}(X_{i}+Y_{i})/2}$$ (1)

where *X*_*i*_ and *Y*_*i*_ are the counts of species *i* in samples *X* and *Y*, respectively. When expressed in this form, the intuitive notion of similarity between samples as the proportion of overlap is manifested through the minimum function. The BC index can be expanded as

$$S=100\frac{\sum_{i}\left[X_{i}^{<}+Y_{i}^{<}+\left(X_{i}^{=}+Y_{i}^{=}\right)/2\right]}{\sum_{i}(X_{i}+Y_{i})/2}.$$ (2)

where the notation $X_{i}^{<}$ ($Y_{i}^{<}$) designates counts for species *i* in sample *X* (*Y*) that are less than the counts in sample *Y* (*X*), and $X_{i}^{=}$ and $Y_{i}^{=}$ are the counts that are equal in both samples. This equation in terms of species counts can be recast into a similarity index for WGS metagenomic sequence in two samples, *X* and *Y* in the following way. We first identify shared *k*-mers in each sample where, for a read of length *L*, all *n*_L_ = *L−k* + 1 overlapping *k*-mers are considered. The read length *L* may be variable if reads are trimmed in connection with quality filtering. For the *j*th read in sample *X* we calculate the sum of the differences

$$Z_{j}^{X}=\sum_{i}\left(x_{ij}^{X}-y_{ij}^{X}\right)$$ (3)

where $x_{ij}^{X}$ ($y_{ij}^{X}$) is the number of times the *i*th *k*-mer occurs in sample *X* (*Y*), and the sum is over all overlapping *k*-mers. The count difference is similarly calculated for all reads in sample *Y*,

$$Z_{j}^{Y}=\sum_{i}\left(y_{ij}^{Y}-x_{ij}^{Y}\right).$$ (4)

In eqs (3) and (4) the sums are only over values of *i* for which both $x_{ij}^{X}$ and $y_{ij}^{X}$ ($x_{ij}^{Y}$ and $y_{ij}^{Y}$) are nonzero. That is, the sum is only over differences in counts for *k*-mers found in both samples. The count differences are constructed such that they are negative where the *k*-mer count is lesser in the sample from which the reads are drawn. When obtaining the *k*-mer counts in the two samples for a given read, it is necessary that the read itself not be included in the count for that *k*-mer. The reason for this is that the probability that a *k*-mer in that read belongs to the sample it is drawn from is unity for every *k*-mer in that read, but the probability of occurrence of this *k*-mer among the other reads in the same sample or the other is binomial. Therefore, the probability distribution of *k*-mer counts is binomial only if we subtract one from $x_{ij}^{X}$ and $y_{ij}^{Y}$, which is equivalent to removing the read being considered from the count. Using indicator functions of the count differences for lesser counts

$$I_{<}(Z)=\{\begin{array}{cc} 1 & Z<0\\ 0 & Z\geq 0 \end{array}$$

and equal counts

$$I_{=}(Z)=\{\begin{array}{cc} 1 & Z=0\\ 0 & Z\neq 0 \end{array}$$

we define the binary variables $X_{j}^{<}=I_{<}(Z_{j}^{X})$, $Y_{j}^{<}=I_{<}(Z_{j}^{Y})$, $X_{j}^{=}=I_{=}(Z_{j}^{X})$ and $Y_{j}^{=}=I_{=}(Z_{j}^{Y})$. We also define the constants, *X*_*j*_ = 1 and *Y*_*j*_ = 1 in order to get the total number of reads in each sample. Substituting these quantities into eq (2) where the sums are now over all reads

$$\begin{array}{ll} S & =100\frac{\sum_{j}\left(I_{<}(Z_{j}^{X})+I_{=}(Z_{j}^{X})/2\right)+\sum_{j}\left(I_{<}(Z_{j}^{Y})+I_{=}(Z_{j}^{Y})/2\right)}{\frac{1}{2}\left(\sum_{j}X_{j}+\sum_{j}Y_{j}\right)}\\ & =100\frac{\sum_{j}\left(X_{j}^{<}+X_{j}^{=}/2\right)+\sum_{j}\left(Y_{j}^{<}+Y_{j}^{=}/2\right)}{\frac{1}{2}\left(\sum_{j}X_{j}+\sum_{j}Y_{j}\right)} \end{array}$$ (5)

we obtain a similarity score for metagenomics reads in two samples. Note that *k*-mer counts are only used as a means of categorizing reads. The similarity score is based on tallying reads. By summing only over reads with shared *k*-mers the contribution from noise due to sequencing error is minimized. The similarity score for WGS metagenomic sequence reads derived by this approach is analogous, but not identical, to the BC index. The traditional BC index used in numerical ecology is a species-level measure of similarity. The similarity score in eq (5) measures similarity of genetic content of the microbiome on the scale of shotgun sequence reads, which are generally much smaller than microbial genes, and is affected by duplicated regions and strain-level differences in sequence.

### Optimization and validation

Optimization of the alignment-free algorithm implemented by GUTSS involves a compromise between biological, statistical and technical constraints. The *k*-mer length influences the ability to uniquely map WGS metagenomic sequence between samples. The percentage of unique *k*-mer sequences with length 11 to 35 bp for four bacterial species, *Francisella tularensis* subsp. *novicida*, *Escherichia coli*, *Pseudomonas aeruginosa* and *Burkholderia pseudomalleii* are shown in S1 Fig. Spurious matches are more prevalent for *k*-mers shorter than about 17 to 20 nucleotides. At the sequence length (*k* = 31) used throughout this study *k*-mer uniqueness was greater than 99.3% for these bacterial species except for *E*. *coli*, which was 97.8%, presumably due to gene duplication. In the other extreme, opportunity for overlap of sequence reads diminishes with increasing *k*-mer length. For a range of *k*-mer lengths between these extremes, variation in the number of *k*-mers mapped between samples is only weakly dependent on *k*-mer length.

The minimum number of sequence reads required per sample depends on the sequencing technology (length of reads), sequence quality and species diversity in the sample. Variation of percent similarity with coverage for WGS metagenomic sequence from three adult (HMP) gut microbiomes with low, average and high Shannon diversity of 0.99 (SRS013215), 2.02 (SRS016267) and 3.25 (SRS017521) are plotted in S2 Fig for pairs of samples ranging from 10,000 to 20 million reads. Greater than 99% similarity was obtained with 20 million reads. For a single bacterial genome, a binomial model can be used to predict similarity as a function of sample size (number of sequence reads). The percent similarity score, *S* for two sets of *N* reads randomly placed in *k* genomic locations is *S* = 100(1 –(1–1/*k*)^*N*^). This model (blue line) accurately predicted similarity of two mutually exclusive random samples of the 4.1 Mb genome of *Acinetobacter baumannii* (blue dots in S2 Fig). At the other extreme, a similar binomial model for a 3.2 Gb *Homo sapiens* genome is shown for reference.

**Fig 1:**
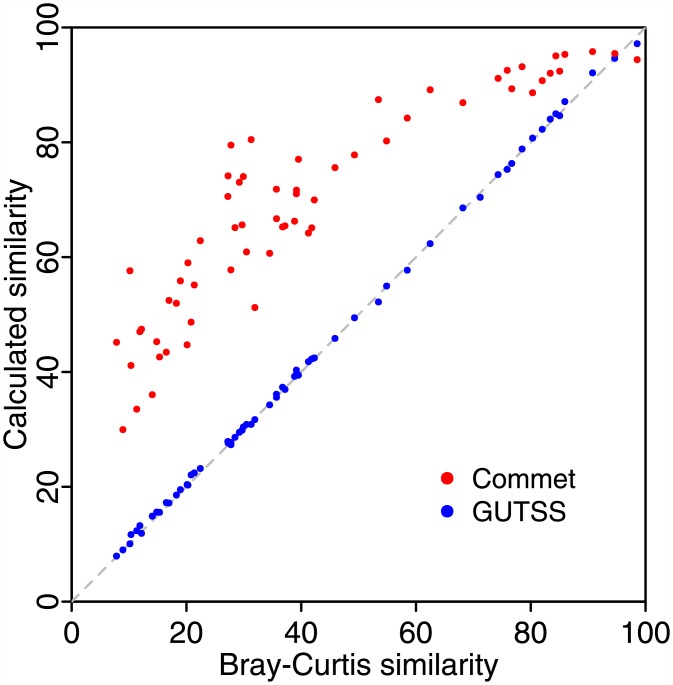
Simulated community analysis of GUTSS reveals sensitivity to relative abundance compared to Commet. Similarity scores for selected pairs of simulated communities (see Methods) using GUTSS and Commet are plotted against BC similarity. The BC index was calculated from the relative abundances used to construct the mock samples. The identity line (grey dashed line) is shown for reference.

Accuracy of the GUTSS method was tested with simulated bacterial communities using whole genome sequence (WGS) reads. Simulated communities of 20 million reads each were generated by random selection of WGS reads from 59 bacteria commonly found in the Human gut microbiome (S1 Table). MetaPhlAn species abundances of the gut microbiomes of donors and patients in an FMT study (see below) were used to simulate realistic abundance profiles. Sequence reads were drawn from among the 59 sequenced genomes to match the abundance profiles (but not the original species). The expected BC similarity of sample pairs was calculated from the species abundances used to construct the mock communities. GUTSS scores for 62 selected sample pairs were plotted against the expected BC similarity (Fig 1). GUTSS similarity deviated from expected values by an average of 0.2±0.6%. In contrast, Commet similarity was higher than BC similarity by an average of 27.4±12.2% (Fig 1).

### Computational performance

The GUTSS software application developed in this study for calculating sample similarity from metagenomic shotgun sequence in fastq file format is available from GitHub [https://github.com/marade/GUTSS]. The Python utility constructs a hash of *k*-mers for all reads in each fastq file using Jellyfish[57], obtains *k*-mer counts for each read using query_per_sequence (slightly modified) from the Jellyfish package, and calculates similarity from total read counts obtained. Similarity scores calculated by GUTSS for two samples with 20 million Illumina reads took 180 minutes on a Intel(R) Xeon(R) CPU E5420 @ 2.50GHz using 5 cores under KVM virtualization. Computation time increases approximately linearly with sample size because most of the computation time involves serially calculating differences in *k*-mers counts for each read, and this scales proportionally with the number of reads. The memory requirement is generally about 1GB per million reads (total in two samples) but varies greatly with system architecture, sample complexity and *k*-mer size.

### Sample cohorts

The pediatric study to treat Crohn's disease with FMT at Seattle Children's Hospital was a single-center open-label study designed to determine tolerability, preliminary safety, and potential efficacy in pediatric patients with CD[47]. Nine patients with mild-to-moderate disease symptoms as defined by Pediatric Crohn’s Disease Activity Index (PCDAI) between 10 and 29, and aged 12 to 21 years, were enrolled into this study. Each participant was followed in the study for approximately 12 weeks. The protocol was approved by the Institutional Review Board of Seattle Children’s Hospital. All patients/participants provided written informed consent or assent. Approval from the FDA (investigational new drug number 14942) was obtained. The study was registered with clinicaltrials.gov [number:NCT01757964]. Study participants were recruited from Seattle Children’s Hospital outpatient gastroenterology clinics.

Children from a cohort of 12 healthy individuals aged between 55 and 1319 days were recruited as controls in a pediatric CF research study at Seattle Children’s Hospital[54]. This study was approved by the Seattle Children’s Hospital Institutional Review Board, and consent was obtained for all subjects. Children in the control group were excluded if they were less than 36 weeks gestation at birth, were under treatment with antibiotics at recruitment or within two months of enrollment, had a known gastrointestinal or immunological disorder or any condition requiring chronic antibiotic therapy, or were acutely ill at time of enrollment. Sample collection and experimental procedure description can be found in the supplementary data for this study[54].

The U.S. National Institutes of Health (NIH) funded Human Microbiome Project (HMP) Consortium adult cohort consists of healthy individuals aged 18 to 40 residing in the U.S.[55]. Stool samples in this study were collected according to the Manual of Procedures and the Core Microbiome Sampling Protocol [available at http://www.hmpdacc.org/tools_protocols/tools_protocols.php]. According to the HMP protocol, Human DNA sequence was identified and removed, duplicate reads were marked and removed, and reads with ambiguous bases were trimmed from each end. WGS metagenomic sequence from 100 gut microbiome samples was downloaded from http://hmpdacc.org/. Twenty samples were randomly selected from the 100 samples for comparison with each of the pediatric study control samples.

### Genomic and metagenomic sequence

The *Acinetobacter baumannii* AB5075-UW sequence generated by Illumina (MiSeq) technology was registered at NCBI under Bioproject PRJNA243297 [Genbank:CP008706, CP008707, CP008708, and CP008709]. Complete genome assemblies were obtained from NCBI GenBank [http://www.ncbi.nlm.nih.gov/genbank/] for *Francisella tularensis* subsp. *novicida* U112 [NCBI: NC_008601.1], *Escherichia coli* O157:H7 TW14359 [GenBank: CP001368.1 and CP001369.1], *Pseudomonas aeruginosa* PAO1 [NCBI: NC_002516.2] and *Burkholderia pseudomalleii* 1026b [NCBI: NC_017831.1 and NC_017832.1]. WGS used to construct simulated communities with accession numbers listed in S1 Table were obtained from the NIH Sequence Read Archive (SRA). Healthy adult gut microbiome WGS metagenomic sequence was obtained from the Human Microbiome Project (http://www.hmpdacc.org/) for the accession numbers listed in S2 Table.

### Sequence processing and data analysis

All metagenomic shotgun sequence used in this study was filtered according to the HMP protocol, including Human sequence removal. Sequence samples were also subjected to a final quality screen in order to remove reads not meeting the requirement of at least 88 of the first 90 bases having a Phred quality score greater than or equal to 20. Commet [https://github.com/pierrepeterlongo/commet] was run with default parameters and *k* = 31. Relative abundance of species was calculated using Metagenomic Phylogenetic Analysis (MetaPhlAn) v2[10]. Bray–Curtis similarity scores derived from species relative abundance were calculated using vegan, a CRAN package for the analysis of ecological communities[58]. For the FMT studies we defined the donor similarity index (DSI) as DSI = (*S*_*t*_−*S*_0_)/(100−*S*_0_) where *S*_0_ is the pre-transplant (baseline) similarity of the recipient to the donor, and *S*_*t*_ is the recipient to donor similarity at a given time point, *t* for the post-transplant samples. All statistical analysis was performed and figures prepared using R (version 3.1, R Development Core Team 2012, R Foundation for Statistical Computing, Vienna, Austria).

## Results

### The gut microbiomes of healthy young children compared to adults

**Fig 2:**
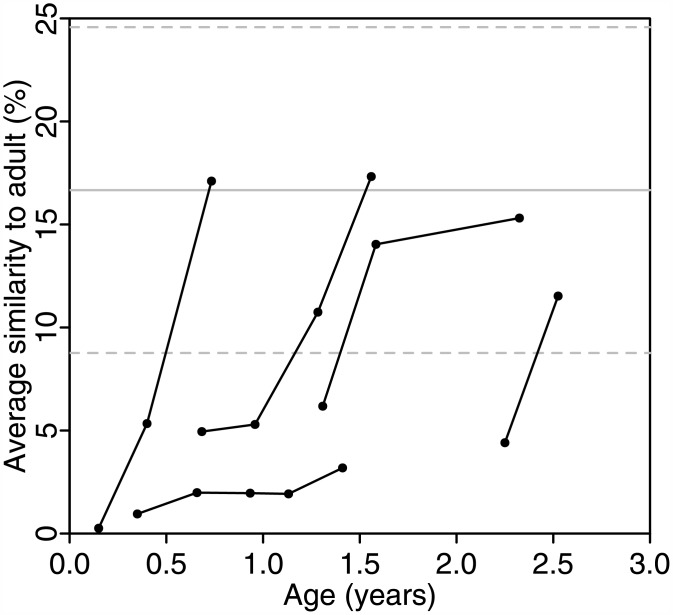
Microbiomes of healthy young children increase in similarity to adults in the first three years. Gut microbiomes of healthy young children serving as controls in a CF study were compared with adult microbiomes from the HMP. The median microbiome similarity (points) for young children compared with a random selection of twenty adults are shown for longitudinal samples (connected by lines) for five young children. The average (solid horizontal line) and first standard deviation (dashed lines) were derived from the 100 adult samples (50 independent pairs).

The gut microbiome of young children up to three years of age is known to be different from that of adults and exhibits greater interpersonal variation[59, 60]. One way to measure how the gut microbiome of a young child progresses toward establishment of an adult-like microbiome is to compare with a pool of healthy adults. We applied GUTSS to compare longitudinal samples from five healthy young children[54] with twenty HMP adult gut microbiomes. The median similarity to adults was determined for each time point (age) of each child (Fig 2). The average similarity of 49 pairs of HMP adult gut microbiomes (solid horizontal line in Fig 2) was estimated by GUTSS to be 16.6±7.5%, with values ranging from 4.8 to 33.6% (S2 Table). Similarity scores for young children ranged from 0.2 to 6% for the first samples taken. Four of the five children were within one standard deviation of the adult average by the time of the last sample. Similarity scores near the adult average were achieved at different timepoints between one and three years of life, suggesting that the establishment of an adult-like microbiome varies across individuals.

### Evaluation of engraftment in FMT therapy

**Fig 3:**
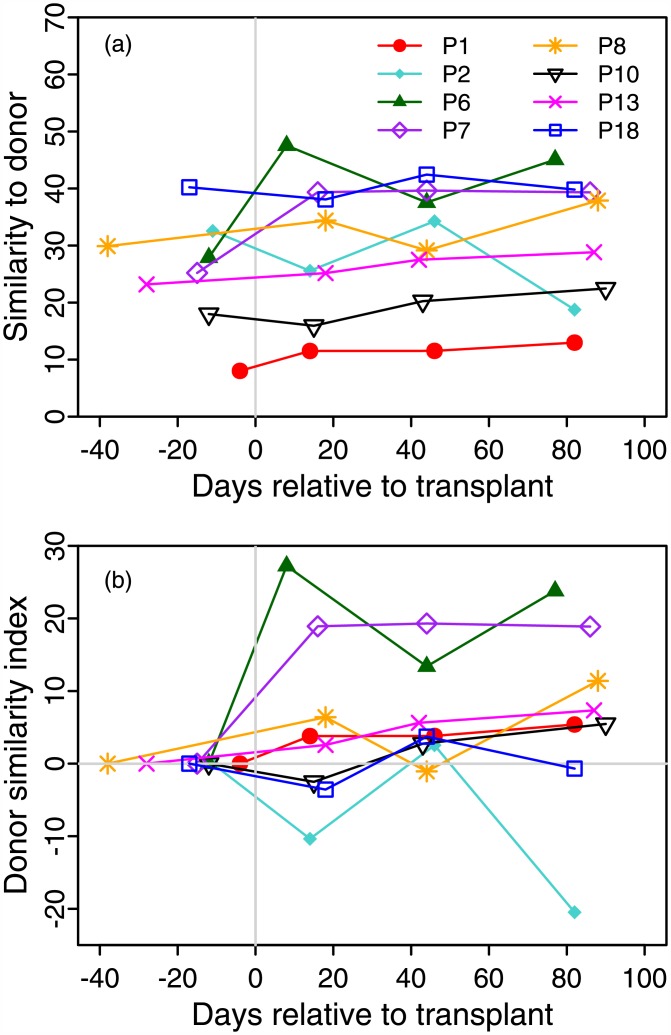
Absolute and relative change in patient similarity to donor is used to track response to FMT. In (a), similarity to baseline donor, pre-FMT and at 2, 6 and 12 weeks following the transplant is shown for eight patients. In (b), the donor similarity index (see Methods) is shown for the eight patients in (a). A large increase in DSI for patients 6 and 7, a mild increase for patients 1, 8 and 13, and no increase (or decrease) for patients 2, 10 and 18 was observed.

WGS metagenomic sequence from patients and donors participating in a pediatric study to treat Crohn's disease with FMT therapy at Seattle Children's Hospital[47] was obtained to detect alteration of the patient's microbiome following transplant. Patients had clinical evaluations and stool collection prior to transplant and at 2, 6, and 12 weeks following FMT. Samples from donors, which were parents of the patients, were acquired on the day of transplant. Samples from eight patients and their respective donors in that study were analyzed using GUTSS. Patient microbiomes were compared with that of their respective donor (Fig 3a). The mean patient to donor similarity, pre-FMT was 25.6±9.7%. Patient 18 had the highest similarity to donor pre-FMT at 40.2%, and Patient 1 had the lowest at 8.0%. The mean similarity to donor was more than one standard deviation above the mean of the adults in the HMP samples, and may be a consequence of relatedness, co-habitation or shared diet of the patients and their donors. Response to FMT therapy is indicated by change from baseline in the recipient's similarity to donor. The highest similarity to donor post-FMT was 47.5% for patient 6, a 19.6% increase from the pre-FMT sample, which is also the largest change observed in this study. Patient 7 had a 14.1% increase to 39.4% following transplant. In contrast, the highest similarity between adult samples in the 100 HMP samples (noted above) was 33.6%. The largest decrease in similarity to donor from baseline was observed for patient 2, from 32.5 to 25.6%. This kind of change can be accounted for by rapid expansion of species not found in the donor, for example.

To compare relative changes in response to FMT therapy, we calculated the donor similarity index (DSI; see Methods). A DSI of 0% represents no change in recipient’s microbiota relative to the donor, a positive score represents the relative percent increase in similarity to donor, while a negative score indicates the relative percent reduction in similarity to the donor’s microbiota following transplant. A DSI of 100% (using our method of calculating similarity) means that the microbial community and their relative abundance are identical in the patient and donor. The DSI at 2 weeks post-FMT revealed a large increase in value for patients 6 and 7, a moderate increase for patients 1, 8 and 13, and no increase for patients 2, 10 and 18 (Fig 3b). All post-FMT scores for four of the patients (1, 6, 7, and 13) were positive. The three patients with negative DSI at two weeks had positive DSI at six weeks. The largest DSI of 27.2% was observed in patient 6 at the two week follow up. The DSI for patient 7 at the three follow up visits, which averaged 18.7±0.3%, was unchanged. The difference between the 2 week and 12 week DSI scores for the five patients with initially positive scores was 1.6±3.5%, though the DSI at 12 weeks ranged 5.4 to 23.8% with mean 13.4±7.8%.

**Fig 4:**
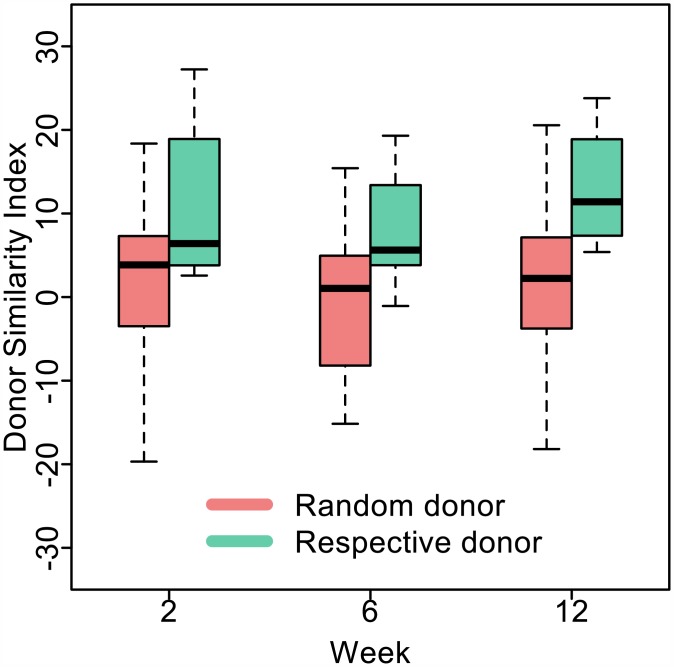
Patient microbiomes became more similar to their respective donors than random donors. Boxplots of the DSI for patients with positive DSI scores compared with their respective donor (green) are shown at 2, 6 and 12 weeks. The boxplots of patients compared with random donors (all donors excluding their own) are also shown (red). The mean DSI for patients compared to their respective donors was 11.8±10.8, 8.2±8.1 and 13.4±7.8, and the DSI when compared to all other donors was 1.6±9.6, -0.7±8.0 and 2.5±8.7 at 2, 6 and 12 weeks, respectively.

Since positive DSI scores suggested successful engraftment of the donor's microbiota in the patient, we sought to determine whether they exceeded similarity scores calculated between patients and random healthy donors, excluding their own. Analysis of variance was used to test whether the patients’ microbiota would become similar to the sampling of healthy donors. Although positive DSI scores were observed for some patients when compared with donors other than their own, the DSI scores were not significantly different from zero at any time point (p > 0.1 at 2, 6, and 12 weeks) (Fig 4). Patients did not on average become more like the random donors (all other donors in this study excluding their own). In contrast, the DSI scores for patients (with positive DSI scores, *n* = 5) compared to their respective donors were significantly greater than the DSI scores with respect to all other donors (at 2 weeks: *p* = 0.035; at 6 weeks: *p* = 0.014; and at 12 weeks: *p* = 0.009) (Fig 4). There were no controls in this trial to determine the inference from these results.

## Discussion

In this study we developed GUTSS, an alignment-free method of WGS metagenomic sequence comparison in order to estimate similarity or changes in microbiomes without having to identify taxonomic constituents, their relative abundances, or phylogenetic relationships. Our similarity score is modeled on the BC index, which is a commonly used ecological measure of community resemblance. By translating this index of similarity for species in two communities into a measure of DNA sequence overlap between two metagenomics samples, we derived an analogous measure that effectively compares microbiomes at the subspecies level. The advantage of measuring microbiome similarity by direct comparison of sequence reads over methods that require species or OTU identification is that it is not biased by incompleteness in our ability to identify microbial species, organism diversity within species, or our knowledge of their function in the human intestine (or other body site). It is robust even with samples containing unculturable microbes. Similarity scores based on species identification generally overestimate similarity where differences at the subspecies level in the actual microbiome communities are not detected. Even similar species can have widely different gene content. Although methods of detection of bacteria in metagenomic samples using marker genes or unique sequences have improved as the number and variety of completed genomes has grown in sequence databases, they will always be limited by incompleteness of the databases, and bacteria will continually adapt to the gut and other environments by gene transfer or deletion.

In order to study changes to the microbiome in clinical research, we used GUTSS to compare gut microbiomes of healthy young children participating as controls in a CF study with that of healthy adults participating in the HMP. Similarity of the young children to the adult average increased toward the adult during the first 18 to 36 months of life. Similar trends have also been previously observed using 16S sequencing and the UniFrac distance measure[59, 60]. We also applied our method to microbiome data in a pediatric study of FMT therapy for patients with CD. Exploratory studies of the potential therapeutic value of FMT in mitigating the effects of inflammatory bowel diseases are dependent upon accurate measures of change in the microbiome of transplant recipients in order to develop correlations with clinical measures of outcome. Recipient to donor similarity prior to transplantation of the microbiota may be an important quantity to track in FMT investigations owing to its potential as a determinant of transplant efficacy. We constructed a DSI score that measures relative change in similarity to donor as a metric for cross-comparison of FMT samples. Three basic responses of large, moderate and no response to transplant were observed (Fig 3b). The relatively larger changes in the microbiome between the pre-FMT and 2 week follow up samples compared with later time points was suggestive that the FMT procedure at least caused a perturbation to the microbiome. Some of this change could be due to effect on the microbiome of the "clean out" preparation prior to transplant. This effect was not controlled for by "placebo" transplants. Since these were not controlled trials, it is not known how the magnitude of these changes compares with normal fluctuations in the microbiome, or whether their cause can be definitively attributed to the transplant procedure. Other factors such as antibiotic therapy, diet or use of probiotics may contribute to observed changes. Even though small samples were used in this trial, it was possible to observe changes in the patients' microbiomes that were significantly increased in similarity to their relative donors compared to all other donors. This suggests that similarity comparison of the metagenomic data using GUTSS is a sensitive test of changes to the microbiome that is useful for clinical evaluation of engraftment.

### Conclusions

Analysis of microbiomes using an alignment-free methodology to calculate similarity analogous to the BC index provides an alternative approach to measuring changes that may be of clinical relevance in studies of the human gut microbiome. We have demonstrated its value for measuring engraftment in FMT therapy studies and its potential for evaluating factors, such as donor to patient similarity, that may be associated with transplant efficacy. Such techniques can also provide more accurate diagnostic assessment of complex human microbiomes to identify patients with an ‘abnormal’ microbiome who might benefit from targeted therapies to alter the microbial community.

## Supporting Information

S1 Fig Percentage of unique *k*-mer sequences for four bacterial species. For further description see Methods.
(PDF)

S2 Fig Variation of percent similarity with coverage for random pairs of samples. GUTSS similarity scores (dots with dashed lines to guide the eye) for 3 HMP adult gut microbiome samples with low, average and high Shannon diversity (SD). Solid lines are models for the *A*. *baumannii* (with dots for GUTSS scores) and *Homo sapiens* genomes. For further description see Methods.
(PDF)

S1 Table Species used to construction the gut microbiome simulated communities and the NIH Sequence Read Archive (SRA) accession numbers. (PDF)

S2 Table HMP gut microbiome metagenomics sample accession numbers and their (paired) similarity scores. (PDF)
