# Supplementary figures and images for: GUTSS: An Alignment-Free Sequence Comparison Method for Use in Human Intestinal Microbiome and Fecal Microbiota Transplantation Analysis
Source: PLoS One. 2016 Jul 8;11(7):e0158897. doi: 10.1371/journal.pone.0158897 (PMC4938407; doi:10.1371/journal.pone.0158897)

Percent unique  $k$ -mers

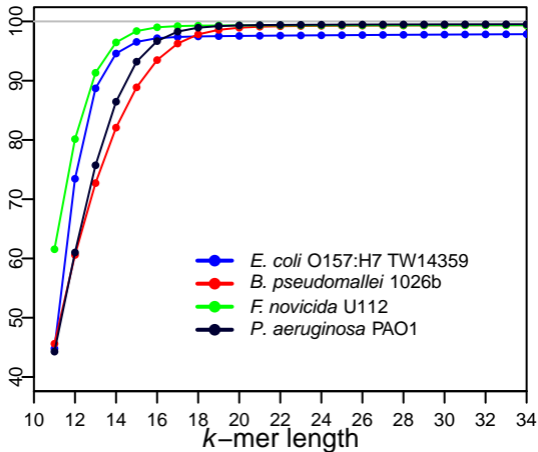

Supplement: S1 Fig — For further description see Methods. (PDF) [file pone.0158897.s001.pdf]

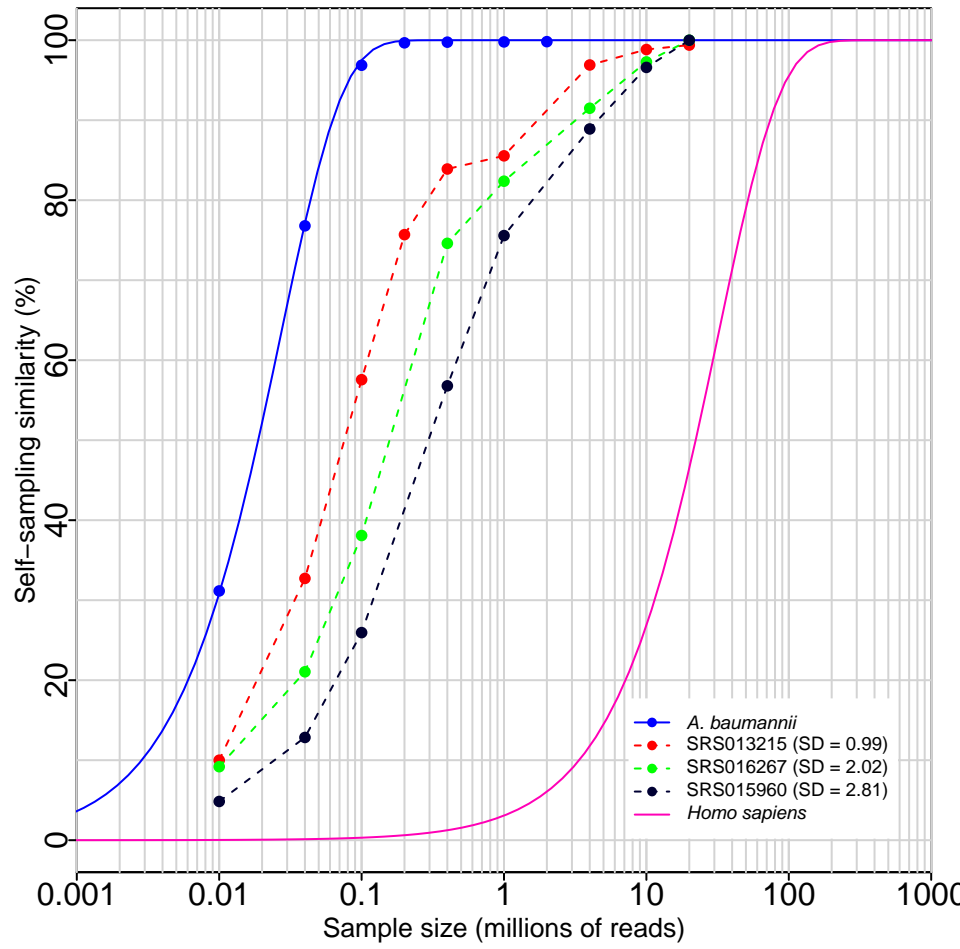

Supplement: S2 Fig — GUTSS similarity scores (dots with dashed lines to guide the eye) for 3 HMP adult gut microbiome samples with low, average and high Shannon diversity (SD). Solid lines are models for the A. baumannii (with dots for GUTSS scores) and Homo sapiens genomes. For further description see Methods. (PDF) [file pone.0158897.s002.pdf]
