# Supplementary material for: GUTSS: An Alignment-Free Sequence Comparison Method for Use in Human Intestinal Microbiome and Fecal Microbiota Transplantation Analysis
Source: PLoS One. 2016 Jul 8;11(7):e0158897. doi: 10.1371/journal.pone.0158897 (PMC4938407; doi:10.1371/journal.pone.0158897)
Supplement: S1 Table — (PDF) [file pone.0158897.s003.pdf]

S1 Table Mock Communities Species Source

Species	Accession
Alistipes shahii	SRR950443
Bacteroides caccae	SRR950446
Bacteroides fragilis	ERR1204056
Bacteroides ovatus	ERR1204053
Bacteroides thetaiotaomicron	SRR3098568
Bacteroides uniformis	ERR1204038
Bacteroides vulgatus	SRR1006216
Bacteroides xylanisolvens	SRR611384
Bifidobacterium adolescentis	ERR1204054
Bifidobacterium bifidum	SRR2088901
Bifidobacterium breve	SRR2088902
Bifidobacterium catenulatum	SRR1151273
Bifidobacterium longum	ERR1308109
Bifidobacterium pseudocatenulatum	ERR1204070
Bifidobacterium pseudolongum	SRR1151283
Bilophila wadsworthia	SRR951543
Blautia producta	ERR171261
Citrobacter freundii	SRR2965635
Clostridium clostridioforme	SRR543903
Clostridium difficile	ERR1204032
Clostridium perfringens	ERR1204026
Clostridium symbiosum	ERR171274
Collinsella aerofaciens	ERR257043
Coprobacillus sp. 8 2 54BFAA	SRR034551
Dorea formicigenerans	ERR1203975
Dorea longicatena	ERR1204063
Enterobacter cloacae	SRR3112323
Enterococcus avium	ERR375594
Enterococcus durans	ERR375599
Enterococcus faecalis	ERR1204025
Enterococcus faecium	SRR3176161
Enterococcus gallinarum	ERR1212000
Enterococcus raffinosus	ERR388711
Escherichia coli	SRR1982079
Eubacterium rectale	ERR043340
Faecalibacterium prausnitzii	SRR088886
Finegoldia magna	SRR2093840
Haemophilus parainfluenzae	SRR387793
Klebsiella oxytoca	SRR3242014
Klebsiella pneumoniae	ERR1335201
Lactobacillus fermentum	ERR570247
Lactobacillus gasseri	ERR1204024
Lactobacillus reuteri	ERR256993
Lactobacillus rhamnosus	ERR1204013
Megasphaera micronuciformis	SRR1518647
Parabacteroides merdae	SRR951882
Peptostreptococcus anaerobius	SRR2095936
Prevotella bivia	SRR2096963
Propionibacterium avidum	SRR2138595
Roseburia intestinalis	ERR043346
Roseburia inulinivorans	ERR257036
Rothia mucilaginosa	SRR835808
Ruminococcus gnavus	ERR1204044
Ruminococcus obeum	ERR043348
Ruminococcus torques	ERR1204060
Streptococcus parasanguinis	ERR716352
Streptococcus salivarius	SRR2143450
Subdoligranulum sp. hmp mda pilot jcvi 0110	SRR866855
Veillonella parvula	ERR1203716
