# Supplementary material for: GUTSS: An Alignment-Free Sequence Comparison Method for Use in Human Intestinal Microbiome and Fecal Microbiota Transplantation Analysis
Source: PLoS One. 2016 Jul 8;11(7):e0158897. doi: 10.1371/journal.pone.0158897 (PMC4938407; doi:10.1371/journal.pone.0158897)
Supplement: S2 Table — (PDF) [file pone.0158897.s004.pdf]

S2 Table. HMP gut microbiome samples and their similarity

HMPsample1	HMPsample2	Similarity
SRS011239	SRS024625	27.3
SRS011405	SRS019968	24.8
SRS011529	SRS023583	15.2
SRS011586	SRS017433	16.0
SRS013215	SRS056519	32.3
SRS014287	SRS019787	7.5
SRS014313	SRS056259	20.3
SRS014613	SRS022071	18.4
SRS014683	SRS015190	24.9
SRS014923	SRS045004	10.8
SRS014979	SRS019267	6.8
SRS015065	SRS016753	15.3
SRS015133	SRS063040	17.5
SRS015217	SRS019030	11.3
SRS015578	SRS052697	19.0
SRS015663	SRS024009	9.9
SRS015782	SRS051031	18.0
SRS015854	SRS014235	18.5
SRS016018	SRS045713	23.5
SRS016495	SRS015960	5.5
SRS017247	SRS017191	33.6
SRS017701	SRS050925	23.6
SRS018351	SRS045645	28.9
SRS018427	SRS019397	21.0
SRS019685	SRS014459	23.1
SRS020328	SRS053214	14.5
SRS020869	SRS016267	8.2
SRS021948	SRS050752	17.2
SRS022713	SRS023526	14.8
SRS023346	SRS012273	8.7
SRS024549	SRS013521	7.2
SRS042628	SRS016335	8.8
SRS043001	SRS012902	21.9
SRS047044	SRS017521	8.4
SRS049712	SRS017821	8.7
SRS049959	SRS016095	8.7
SRS049995	SRS015794	14.9
SRS050422	SRS013476	15.6
SRS051882	SRS054956	14.3
SRS054590	SRS018817	4.8
SRS055982	SRS013800	25.4
SRS057717	SRS063985	9.3
SRS058723	SRS053335	13.0
SRS062427	SRS018575	10.9
SRS064557	SRS019161	32.2
SRS065504	SRS024132	20.9
SRS077730	SRS064276	20.6
SRS078176	SRS019601	9.9
SRS103987	SRS024331	21.5
